# Epstein-Barr Virus Infection in an Elderly Nonimmunocompromised Adult Successfully Treated with Rituximab

**DOI:** 10.1155/2014/641483

**Published:** 2014-02-10

**Authors:** Jacob P. Smeltzer, Matthew T. Howard, Wilson I. Gonsalves, Thomas E. Witzig

**Affiliations:** ^1^Division of Hematology, Department of Medicine, Mayo Clinic, 200 First Street SW, Rochester, MN 55905, USA; ^2^Division of Hematopathology, Department of Laboratory Medicine and Pathology, 200 First Street SW, Rochester, Minnesota, MN 55905, USA

## Abstract

Epstein-Barr virus (EBV) is a ubiquitous virus that commonly affects children and adolescents. In addition to causing a viral illness, it is also associated with various malignancies in particular B cell lymphomas and lymphoproliferative disorders. Differentiating between the two processes can be a diagnostic challenge. Here, we present a case of an atypical EBV infection in an elderly patient with severe systemic symptoms, multiorgan involvement, lymphadenopathy, and negative EBV serology. Excisional lymph node biopsy demonstrated features of a lymphoproliferative process involving EBV. Despite supportive care, she experienced continued clinical deterioration and was successfully treated with rituximab. This case illustrates the diagnostic challenges of these cases particularly in the elderly who may have age related immunosenescence, the utility of EBV PCR testing, and the clinical efficacy of rituximab in clearing the infected cells.

Epstein-Barr virus (EBV) is a ubiquitous virus that afflicts >90% of adults usually as adolescents and young adults. Primary EBV infections in children are often asymptomatic but infections in adolescents can manifest as infectious mononucleosis with overt symptoms of an acute viral infection. After primary infection, EBV typically persists in memory B cells in an asymptomatic latent state [1]. Natural killer cells and CD4+ and CD8+ T cells control EBV-induced B cell proliferation. Various malignancies have been associated with EBV including solid tumors such as nasopharyngeal carcinoma [2]; however, it is most commonly associated with various types of lymphoma including Burkitt's, Hodgkin, HIV related non-Hodgkin lymphoma (NHL), posttransplant lymphoproliferative disorder, and T cell NHL. In addition, EBV positive diffuse large B cell NHL is a recognized type of DLBCL that occurs in the elderly. This entity was first described in East-Asian population [3] and appears less common in western population [4]. EBV infections can also produce lymphadenopathy and systemic symptoms that mimic true lymphoma often presenting a difficult diagnostic and therapeutic dilemma for the clinician. Herein, we describe such a case that illustrates the utility of EBV quantitation by PCR for diagnosis and rituximab for therapy in an immunocompetent female.

A 65-year-old previously healthy female presented to an outside clinic with a one-week history of fatigue, fever, and neck swelling. Past medical history was negative for known prior Epstein-Barr infection or infectious mononucleosis. On examination she was noted to have cervical lymphadenopathy. Initially, laboratory studies demonstrated a leukocytosis of 11.4 × 10^9^/L with lymphocyte predominance and an elevated AST 182 (upper limits of normal (ULN) 43) and ALT of 107 (ULN 45). Serological testings for group B streptococcus, hepatitis A and hepatitis B and EBV were negative. Specifically, the IgG and IgM EBV antibodies were negative consistent with no prior infection. She was felt to have an upper respiratory infection and prescribed supportive care. One week later, her fatigue, fever, and cervical swelling had progressed. On repeat examination, her leukocytosis increased to 25 × 10^9^/L and AST 245 and ALT 285. A CT scan of her chest, abdomen, and pelvis was obtained and it revealed diffuse adenopathy and splenomegaly. She was admitted to a local hospital. Subsequent blood cultures were negative and she continued to decline despite broad spectrum antibiotics. She was transferred to our institution for further diagnostic evaluation.

On arrival, she had defervesced but had persistent fatigue, dyspnea, and nausea. Her complete blood count was notable for a normocytic anemia with a Hgb 9.9 g/dL and a slight lymphocytosis 6000 × 10^9^. Her chemistry profile was consistent with cholestasis with an elevated alkaline phosphatase of 942 (ULN 142), AST 193, ALT 118, and total bilirubin of 1.4 (ULN 1.0). Her LDH was twice the upper limit of normal at 532 (ULN 222). Repeat blood cultures remained negative. Additional microbiology serologies were negative for HIV, Blastomycosis, *Coccidioides*, Histoplasma, *Cryptococcus*, *Brucella*, and Lyme disease. Molecular PCR studies were negative for CMV, HIV, Adenovirus, HHV6, Anaplasmosis, and *Ehrlichia*. A repeat chest X-ray demonstrated bilateral pleural effusions that on thoracentesis appeared bloody and were exudative with 80% lymphocyte predominance but negative for malignancy by cytological exam and flow cytometry. EBV serology was negative on two repeated occasions including EBV VCA IgG, VCA IgM, and EBNA antibody. PET/CT scan demonstrated hypermetabolic adenopathy above and below the diaphragm with diffuse involvement of the spleen. The largest peripheral lymph node was 1.8 cm with SUV max of 3.5. The working diagnosis at this point was lymphoma and a diagnostic excisional lymph node biopsy was obtained. The lymph node was effaced by paracortical expansion of small lymphocytes, larger lymphocytes with nucleoli, plasma cells, histiocytes, and eosinophils. The larger lymphocytes stained positive for CD20 and dim CD30 but were negative for CD10, CD21, BCL-2, and BCL-6. Using probes that recognize EBV virus, encoded RNA demonstrated EBV positive B cells (Figure 1). No clonal immunoglobulin gene rearrangement was detected. The final diagnosis was EBV associated nodal polymorphic lymphoproliferative disease. EBV PCR analysis of the blood was obtained and was positive at 175,000 copies.

The patient was initially treated with supportive therapy and, over the next seven days, her transaminases improved. However, she remained considerably debilitated due to fatigue, malaise, headaches, and nausea. Over the next week, her performance status (PS) deteriorated to being nearly bed bound with PS 4. Repeat EBV PCR remained markedly positive at 115,000. Three days later, she became more confused and disoriented. An MRI showed diffuse pachymeningeal enhancement. A lumbar puncture was obtained with an elevated protein of 76 mg/dL (ULN 35) and cerebrospinal fluid PCR was positive for EBV; cytology for malignant cells was negative. Given the continued clinical deterioration over a total of 30 days since she became ill, we were elected to treat her with four weekly doses of the anti-CD20 monoclonal antibody rituximab at 375 mg/m^2^/dose. The rationale for this choice was that the EBV virus was infecting the B-cells and rituximab is very potent at clearing CD20+ B-cells. Within two days of her first treatment, she had a dramatic clinical recovery. Her lymphadenopathy, confusion, headache, nausea, and fatigue all improved considerably. A follow-up EBV blood PCR five days after her first treatment with rituximab was negative (0 copies). At a follow-up visit, three months later, she had a complete clinical recovery. All her laboratory abnormalities had been resolved and a PET/CT demonstrated complete resolution of the previous FDC avid nodes and spleen. Her EBV PCR remained negative. Repeat serological evaluations of EBV remained negative. Additional follow-up at 1 year showed continued complete remission. Her evaluation showed normal B and T cell quantification, resolution of her previous polyclonal hypergammaglobulinemia, and positive EBV serology for IgG and negative for IgM consistent with seroconversion.

This case demonstrates the diagnostic complexities of an EBV associated disorder. As noted above, EBV has been associated with a variety of different lymphomas and, in some cases it is thought to have a causal role in their development. EBV lymphoproliferative disorders are often restricted to patients with defects in cellular immunity which permits uninhibited growth of EBV-infected cells [5]. Posttransplant EBV associated lymphoproliferative disorders most commonly occur after solid organ transplantation [6] but outside of the transplant setting, they can also occur due to various iatrogenic immunosuppressive therapies [7]. However, our patient was not currently on nor had she ever received any immunosuppressive therapies. The recent approved provisional diagnosis of EBV positive DLBCL of the elderly is defined as an EBV+ clonal lymphoproliferation that occurs in patients >50 years old without any known immunodeficiency [8]. The immunodeficiency of these patients is thought to be senescent or age related exemplified by the median age of 71 and the highest peak in cases occurring after age 90 [9]. Besides an advanced age at presentation, this subgroup is associated with extranodal presentation and aggressive clinical behavior. Nearly 40% failed to achieve a complete remission with cyclophosphamide, doxorubicin, vincristine, and prednisone (CHOP) compared to 9% in an EBV negative control cohort, though the role of immunotherapy with rituximab is unknown [10]. The morphology of these cases varied across a spectrum of polymorphic to large cell lymphoma. Recently, Dojcinov and others described their experience with 122 patients with EBV+ lymphoproliferative disorders with no identified cause of immunosuppression [11]. They described four different histological subtypes ranging from reactive lymphoid hyperplasia (RH) to DLBCL. Clinical outcomes varied across these various subtypes with majority of patients with RH resolving spontaneously while patients whose histology was consistent with diffuse large B-cell lymphoma had dismal outcomes with a median survival of only 25 months. As opposed to cases of EBV associated lymphoma, our patient had clinical and laboratory features that suggested an acute though atypical systemic infection with EBV that did not spontaneously resolve and was rapidly progressive prior to treatment. Though her serology was initially negative, her blood and CSF PCR were positive suggesting an acute infection. She dramatically responded to a course of rituximab with clearance of the virus and achievement of a complete clinical remission. With later follow-up, her serology did demonstrate seroconversion consistent with this being an acute process. Considering her age and lack of exposure to immunosuppressants it raises the possibility that similar to other EBV lymphoproliferative disorders that age related senescence may have contributed to the disease development. In conclusion, this case highlights the diagnostic challenges of various EBV related diseases in an elderly non immunosuppressed patient, the importance of EBV serology and PCR quantification, need for tissue evaluation, and the clinical efficacy of rituximab in eliminating the virus by destroying the infected cell.

## Figures and Tables

**Figure 1:**
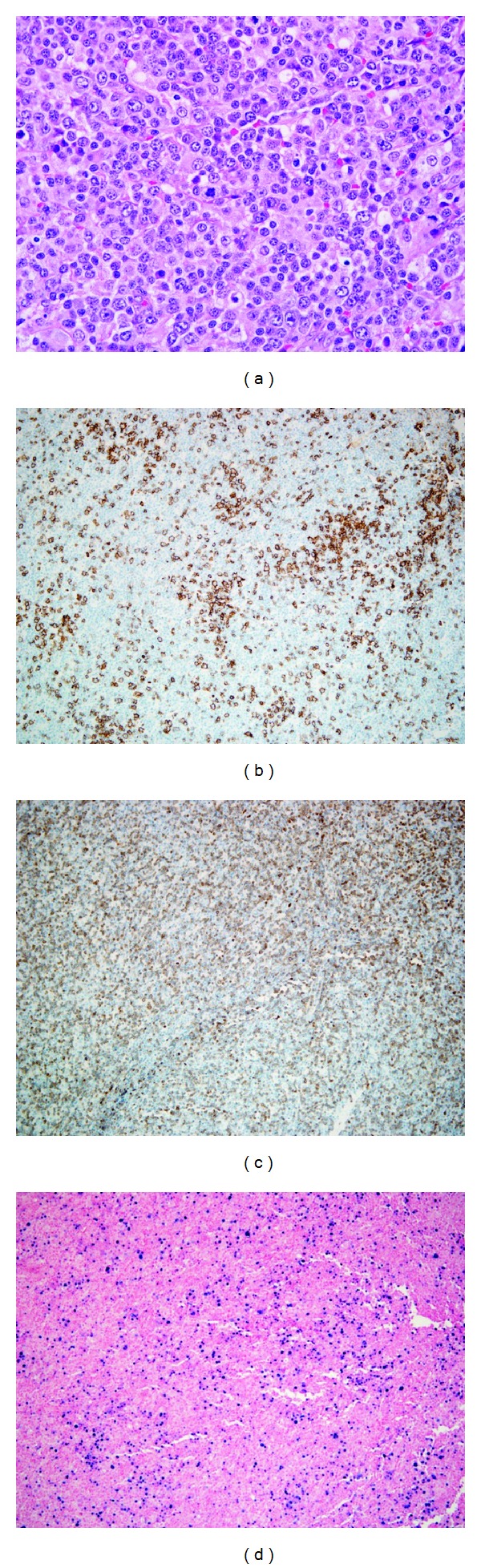
(a) The lymph node is effaced by an infiltrate of lymphocytes with range of cytologic features ranging from large lymphocytes with prominent nucleoli to smaller lymphocytes and plasma cells (hematoxylin and eosin, 400x amplification). (b) Paraffin immunohistochemistry using antibodies against CD20 highlights B-cells with focal loose clusters (CD20, 100x magnification). (c) Small CD3 positive T cells are present in the background (CD3, 100x magnification). (d) Chromogenic in situ hybridization using probes to detect Epstein-Barr virus encoded RNA shows numerous EBV positive cells within the infiltrate, with many more EBV positive cells than would be expected in a lymph node with latent EBV infection (EBV-ISH, 100x magnification).
